# Supplementary material for: Prenatal Bisphenol a Exposure and Postnatal Trans Fat Diet Alter Small Intestinal Morphology and Its Global DNA Methylation in Male Sprague-Dawley Rats, Leading to Obesity Development
Source: Nutrients. 2022 Jun 8;14(12):2382. doi: 10.3390/nu14122382 (PMC9230851; doi:10.3390/nu14122382)
Supplement: Supplementary file 1 [file nutrients-14-02382-s001.zip › nutrients-1712476-supplementary.pdf]

Table S1 Nutrient composition of normal versus trans fat diet. Normal diet formulation was adapted from Mohamed et al., 2012 and Lim et al., 2016.

Ingredient	Normal diet	Trans fat diet
**Carbohydrate**	64%	55%
**Protein**	27%	20%
**Fat**	9%	25%
**Energy (kcal/gram)**	3.9	4.2
Choline	Trace	2g
Folic acid	Not detected	0.002 g
Zinc	Trace	0.0112 g
Vitamin B12	Trace	0.01 g
